# Moving for relief: a meta-analysis on traditional Chinese exercise and nonspecific low back pain

**DOI:** 10.3389/fpain.2026.1729225

**Published:** 2026-04-20

**Authors:** Zixiang Jin, Qianqian Zhang, Meng Wang, Xianglong Zhai, Jiaxin Xi, Yingfei Bai, Jiajia Sang, Hui Ye, Xuezhi Zhang

**Affiliations:** 1 Department of Integrated Traditional Chinese and Western Medicine, Peking University First Hospital, Beijing City, China; 2 Institute of Integrated Traditional Chinese and Western Medicine, Peking University, Beijing City, China; 3 School of Traditional Chinese Medicine, Xinjiang Medical University, Urumqi City, China; 4 Institute of Basic Theory of Chinese Medicine, China Academy of Chinese Medical Sciences, Beijing City, China; 5 Jiangsu Provincial Hospital of Chinese Medicine, Nanjing City, China

**Keywords:** meta-analysis, nonspecific low back pain, pain intensity, Tai Chi, traditional Chinese exercise

## Abstract

**Objective:**

A meta-analysis was conducted to evaluate the efficacy of Traditional Chinese Exercise in reducing pain intensity, improving dysfunction and quality of life, and increasing the overall treatment effectiveness rate in patients with nonspecific low back pain (NLBP), so as to provide evidence for its clinical application.

**Methods:**

Following the PRISMA guidelines, this study systematically searched eight databases—CNKI, Wanfang, VIP, Sinomed, PubMed, Embase, the Cochrane Library, and Web of Science—for randomized controlled trials published up to January 19, 2026. A total of 38 studies involving, 3,054 patients were included. The Cochrane Risk of Bias Tool was used to evaluate the quality of the included studies. Based on heterogeneity, either a fixed-effects or random-effects model was selected to synthesize the standardized mean difference (SMD) or weighted mean difference (WMD), Subgroup analysis and meta-regression were performed to explore the sources of heterogeneity.

**Results:**

The meta-analysis demonstrated that traditional Chinese exercise significantly reduced pain intensity [WMD = −1.21; 95% CI (−1.66, −0.76)], improved dysfunction [WMD = −6.80; 95% CI (−10.18, −3.41)], and enhanced quality of life [SMD = 1.64; 95% CI (0.33, 2.96)] in patients with nonspecific low back pain (NLBP). The overall treatment response rate was increased by 17% [RR = 1.17; 95% CI (1.10, 1.24)]. Subgroup analysis indicated that Liu Zi Jue (a specific Qigong breathing exercise) demonstrated the greatest analgesic effect (SMD = −1.71) and the most significant improvement in functional disability (WMD = −13.35). Additionally, patients younger than 45 years of age showed a more favorable response to treatment (SMD = −1.52). Substantial heterogeneity was observed across studies, attributed primarily to the diversity of intervention protocols, including variations in exercise types, treatment durations, and differences in the measurement instruments used.

**Conclusion:**

Traditional Chinese exercise is an effective, low-cost, and safe intervention for NLBP patients and may be recommended as a complementary approach in clinical management. Future research should include large-sample, standardized studies incorporating biomechanical and imaging techniques to elucidate underlying mechanisms, and develop evidence-based practice guidelines.

**Systematic Review Registration:**

identifier: CRD42025649466 (https://www.crd.york.ac.uk/PROSPERO/).

## 1 Introduction

According to the statistics of the WHO, in 2020, 619 million people worldwide suffered from low back pain. It is estimated that driven by population expansion and aging, the number of patients will increase to 843 million by 2050 (1). Nonspecific low back pain (NLBP) is the most common form of low back pain, accounting for approximately 90% of all cases (2). The pathophysiological mechanisms underlying NLBP remain incompletely understood, and its diagnosis is based on the exclusion of other spinal pain disorders with well-defined diagnostic criteria (3). NLBP is the leading cause of disability worldwide, affecting over numorous people annually, resulting in 64.9 million years lived with disability (4), and incurring a tremendous medical burden and economic cost. Approximately 20% of the global population is affected by NLBP (5), with a recurrence rate of 24%–80% within one year, making it a major driver of workforce loss (6). The lifetime prevalence of low back pain is estimated to range from 50% to 80% (7). About 40% of patients report reduced recreational activities due to pain, and 20% experience impaired daily living functioning, collectively contributing to substantial societal productivity loss. Consequently, NLBP represents a global public health priority for intervention. However, the management of NLBP remains challenging due to the limitations of existing treatment plans and high recurrence rates (8). The mainstream treatments for NLBP include pharmacological approaches such as nonsteroidal anti-inflammatory drugs and physical therapies like spinal surgery (9). However, the long-term dependency on medication and the high recurrence rate following surgery limit their clinical application (10). In recent years, traditional Chinese exercises (TCE) have gained increasing attention as an intervention for NLBP due to their convenience, cost-effectiveness, safety, efficacy, and high applicability (11). These exercises were supposed to enhance core muscle strength, improve blood circulation, regulate psychological and emotional states, and simultaneously address both biomechanical and psychosocial factors associated with low back pain. Several studies have focused on the interventional effects of TCE on low back pain. Li et al. conducted a systematic review and meta-analysis on the efficacy of Baduanjin, which included 9 studies involving 519 patients. The results indicated that Baduanjin made effects on alleviating low back pain (12). Another meta-analysis by Zhang et al., which included 11 randomized controlled trials (RCTs), found that traditional exercises such as Tai Chi and Qigong significantly improved pain intensity (Hedge's g = –0.64) and dysfunction (Hedge's g = –0.96) in patients with low back pain (13). Current research predominantly focuses on single forms of exercise such as Baduanjin or Tai Chi, lacking a comprehensive evaluation of the overall efficacy of traditional Chinese exercises. TCE—including Tai Chi, Baduanjin, Wuqinxi, Yijinjing, and Liuzijue—share a common philosophical foundation rooted in traditional Chinese medicine (TCM) theory, emphasizing the regulation of qi, balance of yin and yang, and harmony between body and mind (14). They also share common physiological mechanisms, including enhancing core muscle strength and spinal stability, improving local blood circulation and anti-inflammatory capacity, and modulating pain perception through mind–body integration (15). Furthermore, these exercises are characterized by similar movement patterns, such as slow, controlled motions, coordinated breathing, and focused mental attention. These shared theoretical underpinnings, biomechanical properties, and therapeutic targets provide a strong rationale for evaluating them as a unified intervention category (16). Aggregating evidence across modalities allows for a more robust assessment of the overall efficacy of TCE, while subsequent subgroup analyses can explore modality-specific effects.

Despite these contributions, prior meta-analyses in this field exhibit several methodological limitations that constrain their clinical applicability. These include a narrow focus on single exercise modalities, limited sample sizes, insufficient handling of heterogeneity across studies, lack of subgroup analyses based on clinically relevant factors (e.g., disease duration, age, treatment frequency), and failure to incorporate recent high-quality randomized controlled trials. Consequently, the existing evidence base is both fragmented and outdated, lacking a comprehensive evaluation of the overall efficacy of TCE as a holistic intervention category. With in-depth investigations into the pathological mechanisms of nonspecific low back pain, there is an urgent need to integrate more comprehensive evidence to clarify the therapeutic effects of TCE on such patients. Therefore, this study expanded the search scope to include a broader range of RCTs, specifically targeting populations with nonspecific low back pain, in order to provide updated and more precise evidence-based support for clinical practice.

## 2 Methods

This report was designed and conducted in accordance with the Preferred Reporting Items for Systematic Reviews and Meta-Analyses (PRISMA) guidelines (17). The meta-analysis was registered on the PROSPERO website (Registration ID: CRD42025649466).

### 2.1 Search strategy

To ensure a comprehensive collection of all relevant literature, a systematic and thorough search strategy was developed. The search will cover the following electronic databases: China National Knowledge Infrastructure (CNKI), Wanfang Database, VIP Database, Chinese Biomedical Literature Database (SinoMed), PubMed, Embase, the Cochrane Library, and Web of Science. The search period spans from the inception of each database to January 19, 2026, and the language is restricted to Chinese and English. The search strategy combines subject headings (such as MeSH terms in PubMed) with free-text terms, utilizing Boolean operators to maximize both sensitivity and specificity, thereby ensuring comprehensive coverage of various keywords and interventions related to LBP. Furthermore, to minimize omissions, the reference lists of included studies as well as bibliographies of relevant systematic reviews and meta-analyses will be manually screened. The detailed search strategy is provided in Supplementary Material S1.

### 2.2 Literature screening

Inclusion Criteria:

1. Study population: Patients with nonspecific low back pain (diagnosis was based on exclusion of other spinal pain disorders with established diagnostic codes (18);
2. Intervention: Traditional Chinese exercises (e.g., Qigong, martial arts, Tai Chi, Baduanjin, Wuqinxi, Yijinjing, Liuzijue) either alone or in combination with other treatment plans; Control: Interventions that did not include traditional exercises compared with the trial group (e.g., usual care, waitlist, health education, conventional physical therapy, etc.);
3. Study type: Randomized controlled trials;
4. Reporting of at least one of the following outcome measures:
  (i) Pain intensity (Visual Analog Scale [VAS], Visual Analog Pain Scale [VAPS], Pain Self-Efficacy Questionnaire [PSEQ], Short-Form McGill Pain Questionnaire [SF-MPQ]), pain degree;
  (ii) Dysfunction index [Roland–Morris Disability Questionnaire [RMDQ], Oswestry Disability Index [ODI], ICF-RS dysfunction];
  (iii) Effective rate (the proportion of patients whose condition improved or reached expected goals under a specific treatment plan);
  (iv) Proprioception [Stability Index [SI], Ankle Force sense Variability [AFV], Average Tracking Error [ATE]];
  (v) Quality of life [Japanese Orthopaedic Association Scores [JOA], Short Form-36 Health Survey [SF-36]];
  Disability [Pain Disability Index [PDI], Quebec Back Pain Disability Scale [QBPDS]];
  Distress (distress level).

Exclusion Criteria:

1. Reviews, case reports, study protocols, or conference papers;
2. Animal and *in vitro* studies;
3. Duplicate publications or articles for which full text could not be retrieved;
4. Studies from which outcome data could not be extracted.

Two reviewers independently screened the literature according to the above criteria. Disagreements during the screening process were resolved through discussion or by consultation with a third reviewer.

### 2.3 Data extraction and quality assessment

Two researchers independently extracted data from the ultimately included studies, including the first author, year of publication, country, randomization and blinding design, intervention and control measures, duration of treatment, sex, age, disease duration, and outcome indicators. The risk of bias in the included randomized controlled trials was assessed across the following five domains: The risks of bias assessed included bias arising from the randomization process, bias due to deviations from intended interventions, bias due to missing outcome data, bias in outcome measurement, and bias in selective reporting of results using the Cochrane Risk of Bias tool (RoB 2.0) (19). For each study, two researchers independently evaluated the quality by rating each of the abovementioned five domains as “low risk,” “high risk,” or “some concerns.” Discrepancies were resolved through discussion or by consulting a third reviewer. The evaluation results are presented in a risk of bias graph.

### 2.4 Data integration and statistical analysis

The primary outcome measures of concern were pain intensity, dysfunction and effective rate, while the secondary outcome measures were proprioception and quality of life. Meta-analysis was conducted using stata15.0. For continuous data, when the same scale is used, calculate the weighted mean difference (WMD) and report the 95% confidence interval (CI). If the trials under evaluation use different scales to measure the same outcome, the standardized mean difference (SMD) with a 95%CI is used to synthesize the data. For binary variables, RR was used as an effect indicator for meta-analysis. The heterogeneity between the studies was evaluated based on the Q test of *Χ*2 and the I2 statistic. Among them, the I2 statistic is an important indicator of heterogeneity, with values of 25%, 50%, and 75% representing low, medium, and high heterogeneity respectively (20). If no significant heterogeneity was observed among the studies (i.e., *I*^2^ < 50% and *P* > 0.1), a fixed-effects model (Mantel–Haenszel method) was employed for the meta-analysis; otherwise, a random-effects model (DerSimonian–Laird method) was used. Subgroup analyses and meta-regression were conducted based on disease duration, age, sex, treatment duration, and type of conventional exercise to identify the magnitude and sources of heterogeneity across studies. Sensitivity analysis was performed to evaluate the robustness of the meta-analysis results. Funnel plots were generated to assess potential publication bias, and Egger's or Begg's test was applied for statistical evaluation when at least five studies were included. For outcomes with significant publication bias, the trim-and-fill method was used to estimate its impact on the results.

## 3 Results

### 3.1 Literature screening results and flowchart

**Figure 1:**
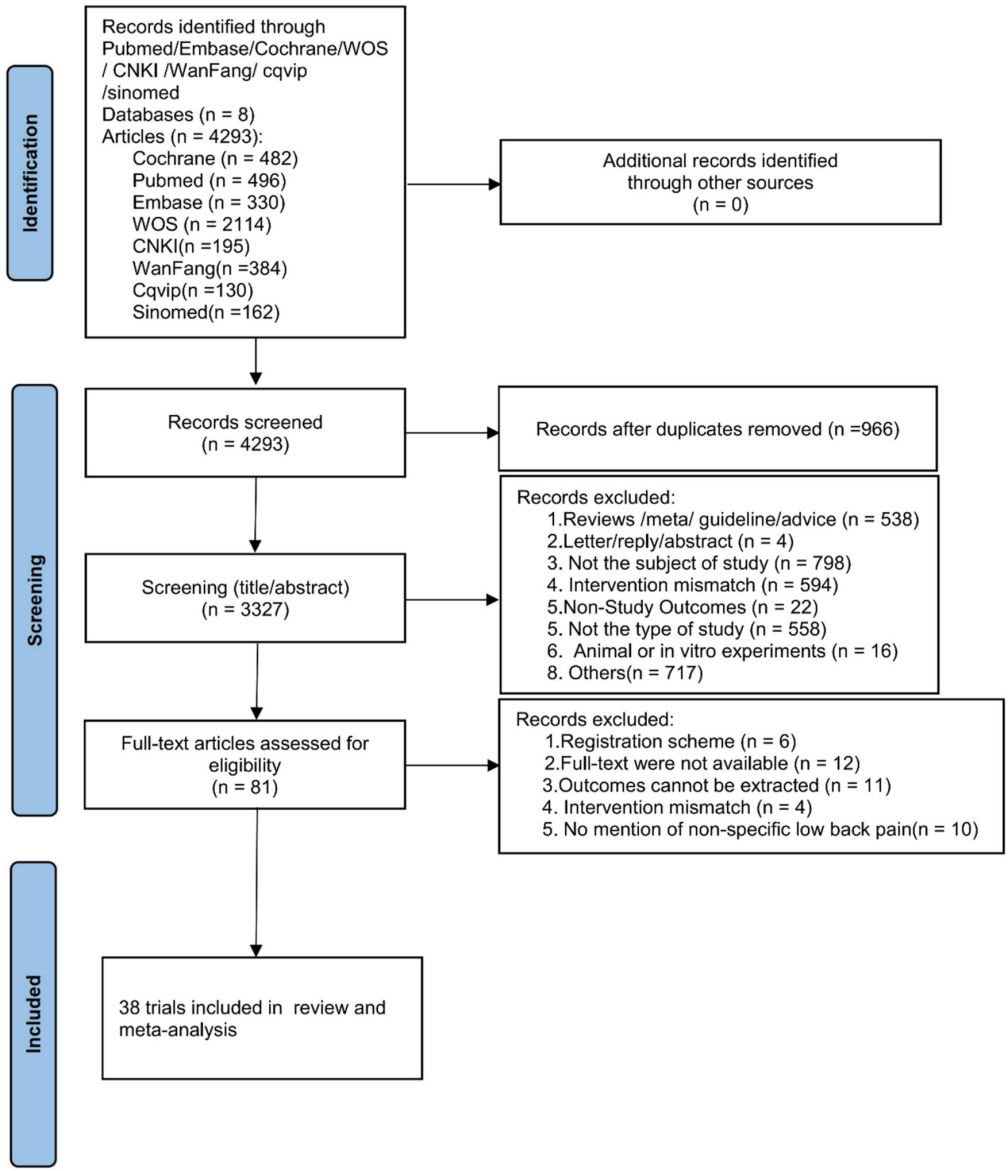
Flowchart of literature screening.

A total of 4,293 articles were initially retrieved from the database search, and no additional studies were identified through reference scanning. After removing duplicates, 3,327 articles were screened based on titles and abstracts. Among these, 3,246 articles were excluded for not meeting the inclusion criteria, and 81 articles underwent full-text review. Ultimately, 38 studies were included in this meta-analysis (21–58) (Figure 1).

### 3.2 Basic characteristics of included studies

A total of 38 studies from three countries [China (*n* = 36), Greece (*n* = 1), and Thailand (*n* = 1)] were included, involving 3,054 patients. The mean age ranged from 21.05 to 70.74 years. Detailed characteristics of the included studies are presented in Supplementary Material S2.

### 3.3 Quality assessment

#### 3.3.1 ROB2 risk of bias assessment

**Figure 2:**
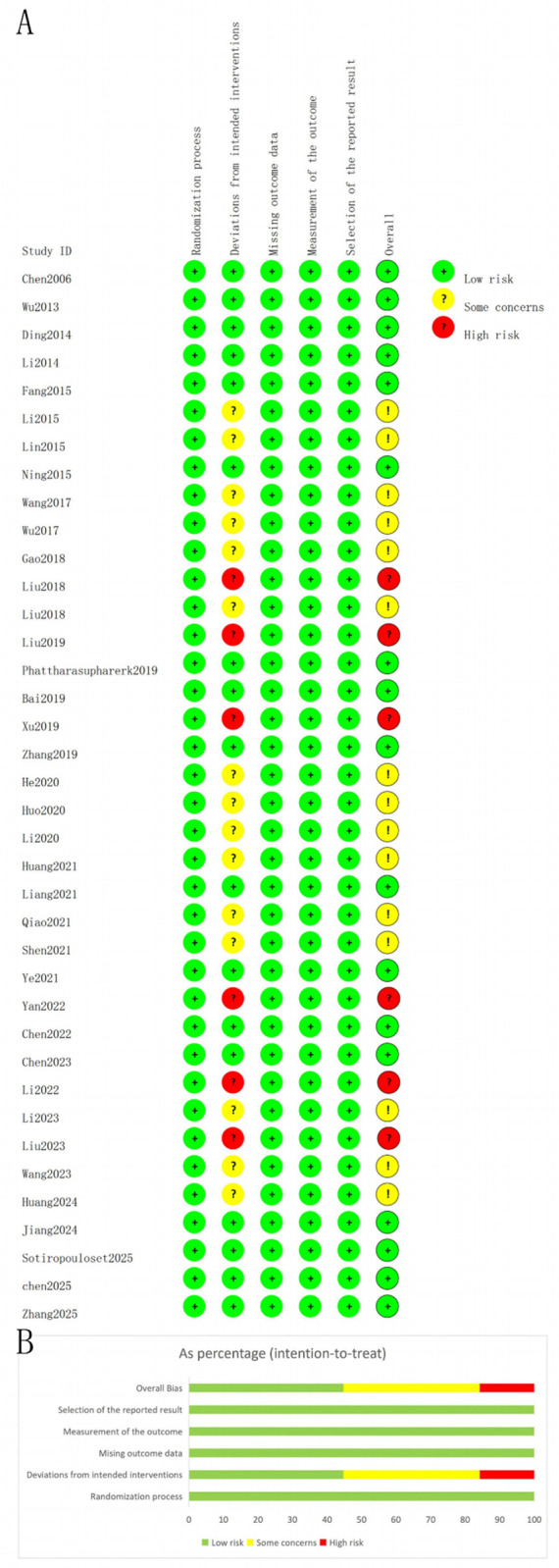
Risk of bias plot. **(A)** Detailed risk of bias assessment for each included study; **(B)** Summary of risk of bias presented as percentages across all studies.

The results of the risk of bias assessment for the 38 included studies are presented in Figure 2. In the domain of bias due to deviations from the intended interventions, 15 studies were judged to be at “some concerns” because deviations from the predefined interventions were likely to have influenced the outcomes. 6 studies were rated as “high risk” due to imbalanced deviations from the predefined interventions across study groups. The remaining 17 studies were classified as “low risk.” All studies were assessed as “low risk” in the domains of randomization process, missing outcome data, and measurement of the outcome. All studies were rated as having “some concerns” regarding the selection of the reported result due to insufficient information. Overall, the included studies exhibited a relatively low risk of bias

### 3.4 Meta-Analysis results

#### 3.4.1 Pain intensity

**Figure 3:**
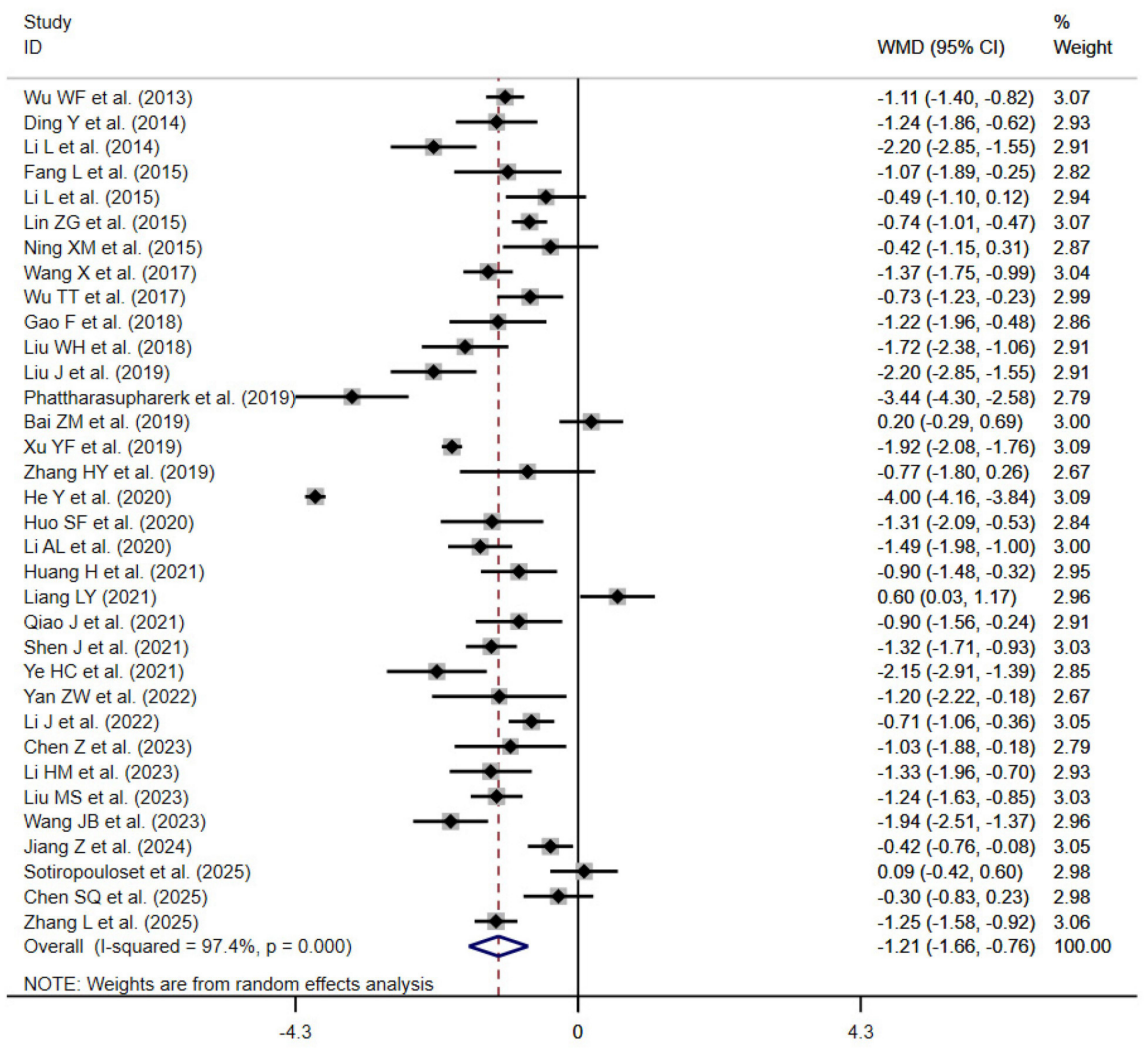
Pain intensity forest plot.

A total of 34 randomized controlled trials (22–31, 33–47, 49–53, 55–58) evaluated the effect of traditional Chinese exercise on pain intensity. The pain intensity outcome was mainly measured using the Visual Analogue Scale (VAS) across the included trials. Meta-analysis using a random-effects model revealed that traditional Chinese exercise significantly improved pain compared with control groups, with a statistically significant difference [WMD = −1.21; 95% CI (−1.66, −0.76); *P* < 0.001; *I*^2^ = 97.4%, *P* < 0.001] (Figure 3).

#### 3.4.2 Dysfunction

**Figure 4:**
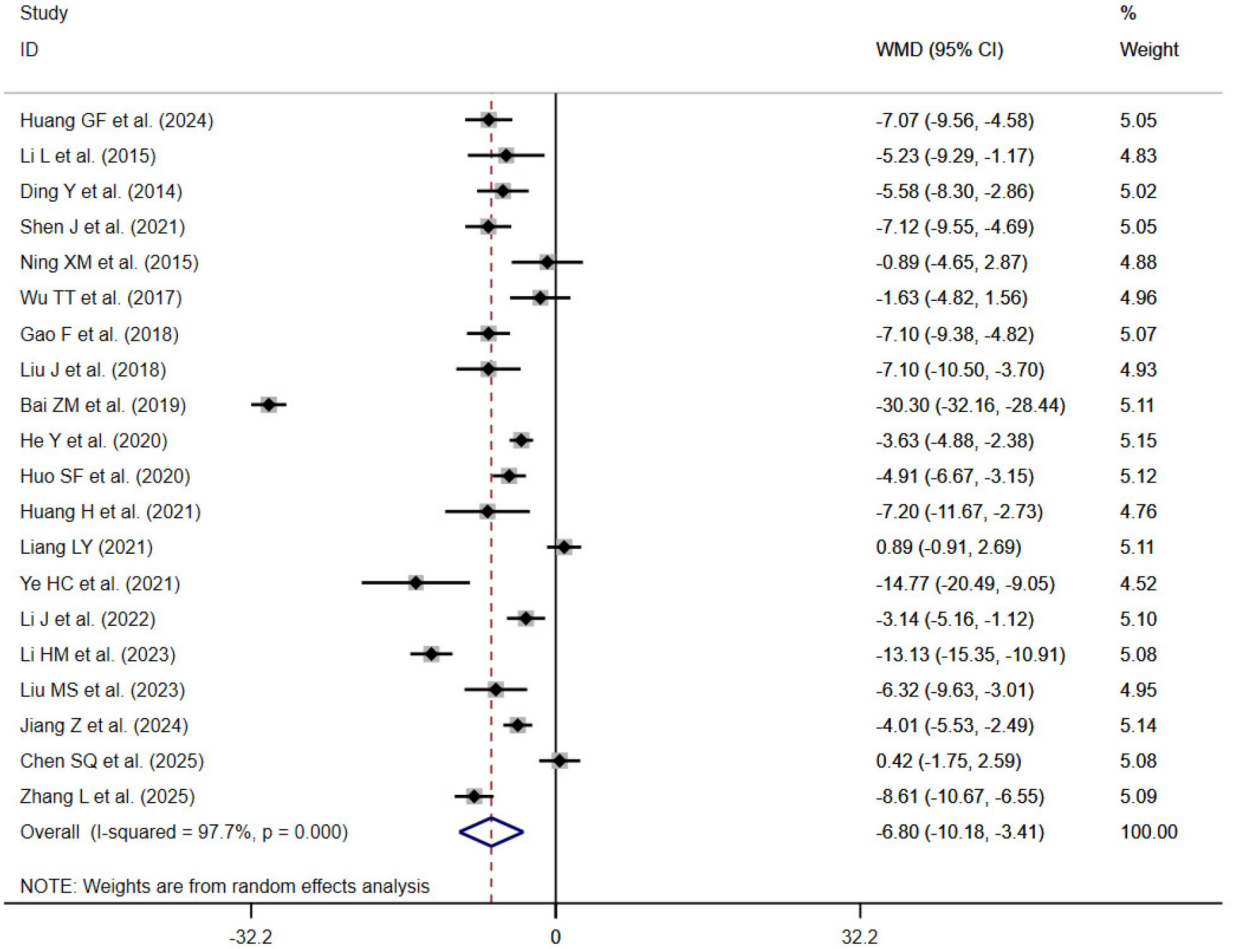
ODI forest plot.

20 studies reported the Oswestry Disability Index (ODI) (21, 23, 26, 28, 30, 31, 34, 36, 39, 40, 42, 43, 46, 49, 51, 52, 54, 55, 57, 58). Significant heterogeneity was identified among the included studies according to heterogeneity testing (*I*^2^ = 97.7%, *P* < 0.001); therefore, a random-effects model was applied for the synthesis of results. The meta-analysis demonstrated that, compared with the control group, traditional Chinese exercise significantly reduced dysfunction in patients [WMD=−6.80; 95% CI (−10.18, −3.41); *P* < 0.001] (Figure 4).

#### 3.4.3 Treatment effective rate

**Figure 5:**
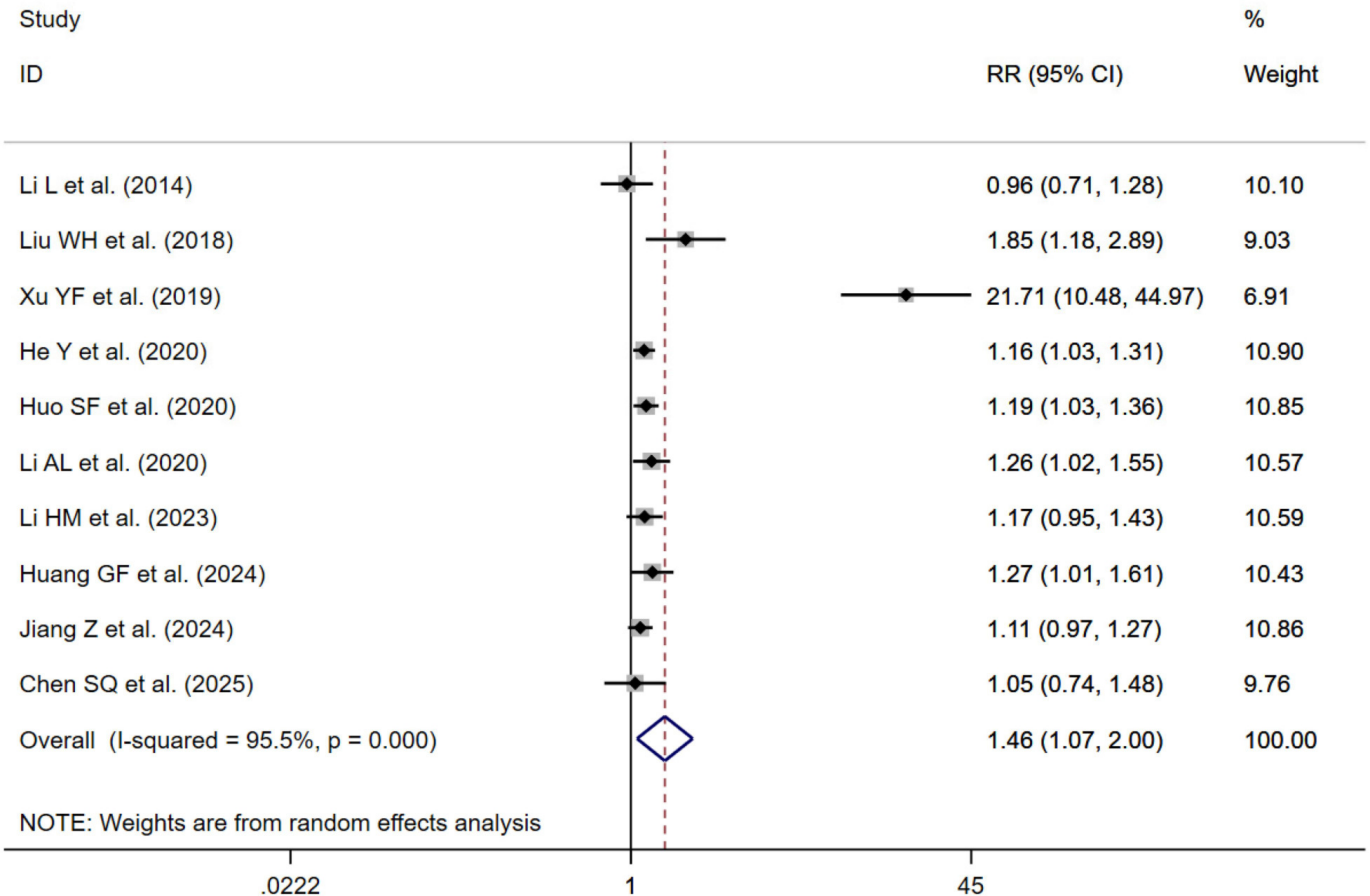
Forest plot of the treatment effective rate (Xu was not deleted).

**Figure 6:**
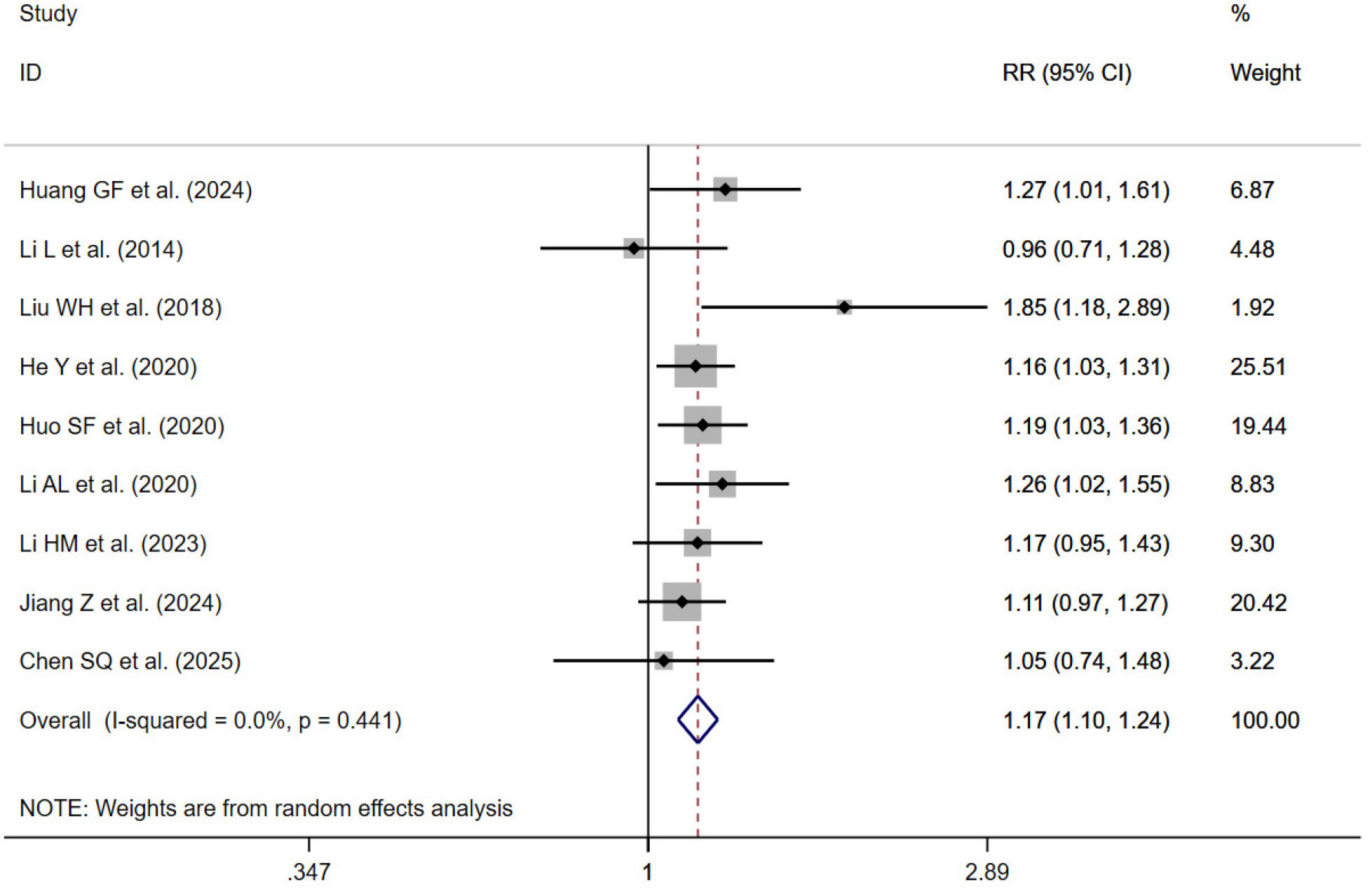
Forest plot of the treatment effective rate (Xu deleted).

10 studies reported treatment effective rate (24, 33, 37, 39–41, 51, 54, 55, 57). Sensitivity analysis revealed that one study (37) exerted a substantial influence on the overall results (Figure 5). After its exclusion, the remaining nine (24, 33, 39–41, 51, 54, 55, 57) studies were synthesized using a fixed-effects model. The results indicated that, compared with the control group, traditional Chinese exercise improved treatment effective rate [RR = 1.17; 95% CI (1.10, 1.24); *I*^2^ = 0.0%; *P* < 0.001] (Figure 6).

#### 3.4.4 Quality of life

**Figure 7:**
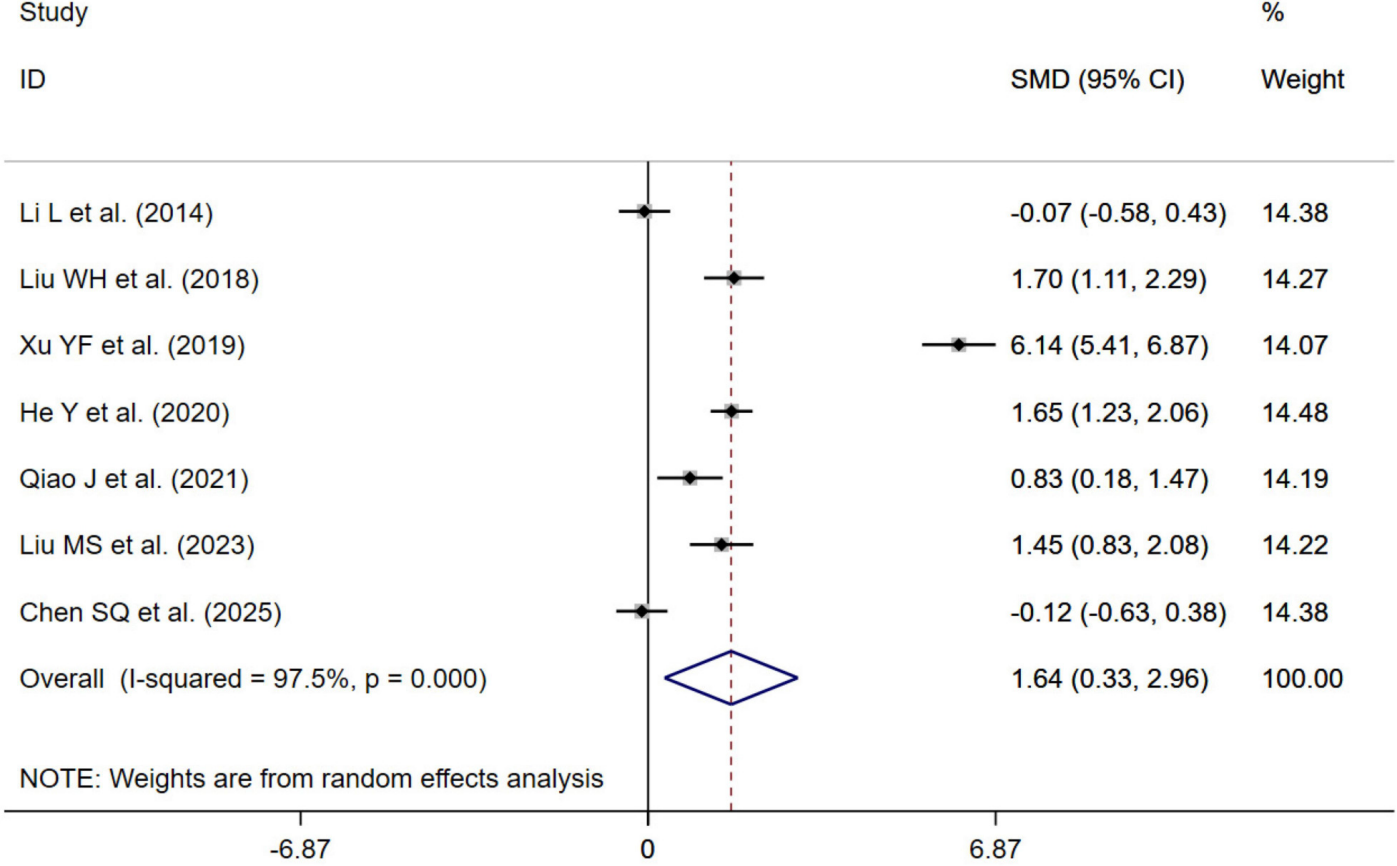
Forest plot of quality of life.

7 randomized controlled trials (24, 33, 37, 39, 44, 52, 57) evaluated the effect of traditional Chinese exercise on quality of life. Meta-analysis using a random-effects model demonstrated that traditional Chinese exercise significantly improved quality of life compared with the control group [SMD = 1.64; 95% CI (0.33, 2.96); *P* < 0.001; *I*^2^ = 97.5%, *P* = 0.015] (Figure 7).

#### 3.4.5 Proprioception

**Figure 8:**
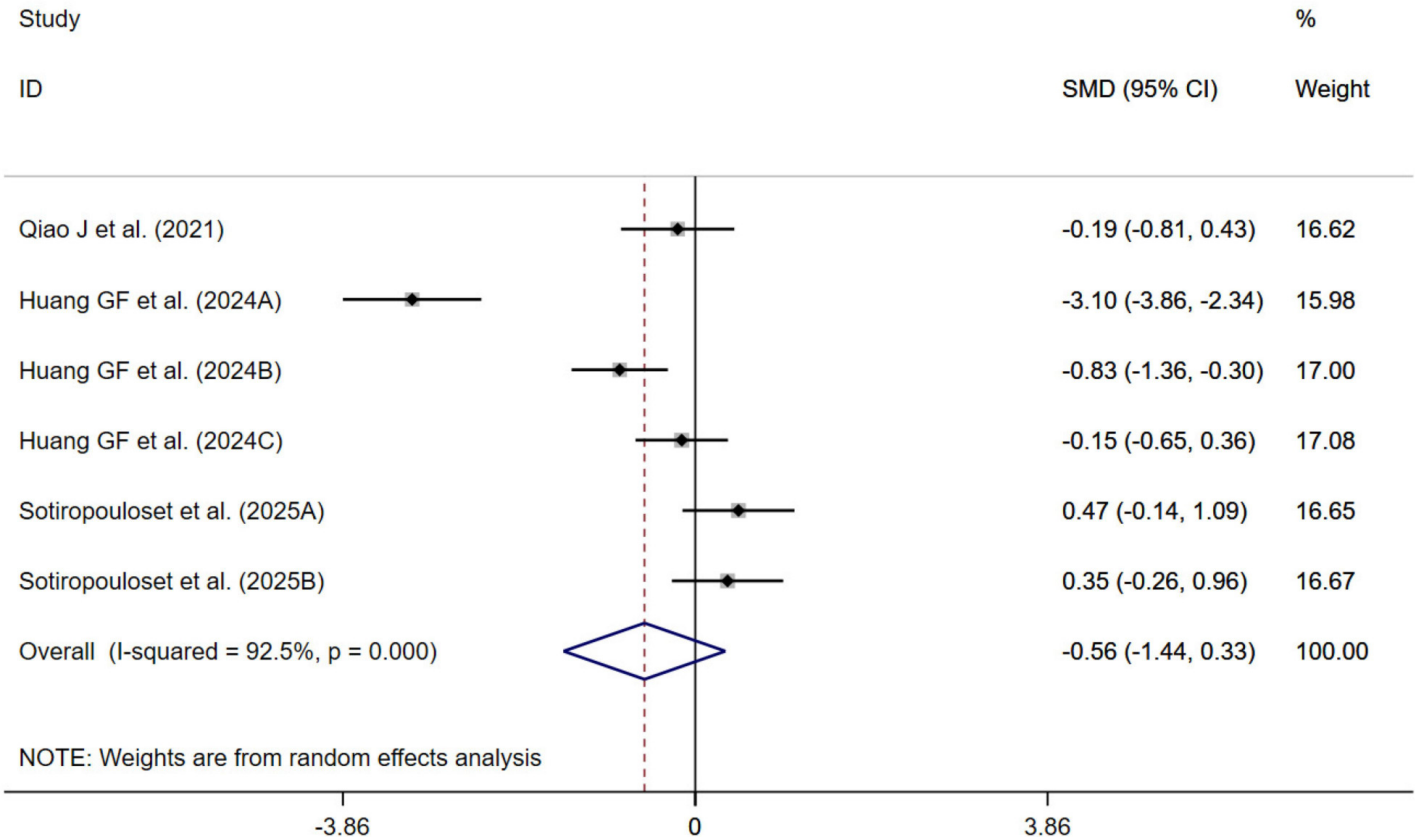
Forest plot of proprioception. In Huang Guofang (54), three assessment methods were adopted to evaluate proprioception. In Sotiropoulos et al. (56), two assessment methods were adopted to evaluate proprioception.

3 randomized controlled trials (44, 54, 56) evaluated the effect of traditional Chinese exercise on proprioception. A meta-analysis using a random-effects model showed that traditional Chinese exercise reduced proprioception compared with the control group, although the difference was not statistically significant [SMD = −0.56; 95% CI (−1.44, 0.33); *P* = 0.219; *I*^2^ = 92.5%, *P* < 0.001] (Figure 8).

#### 3.4.6 Spine range of motion

**Figure 9:**
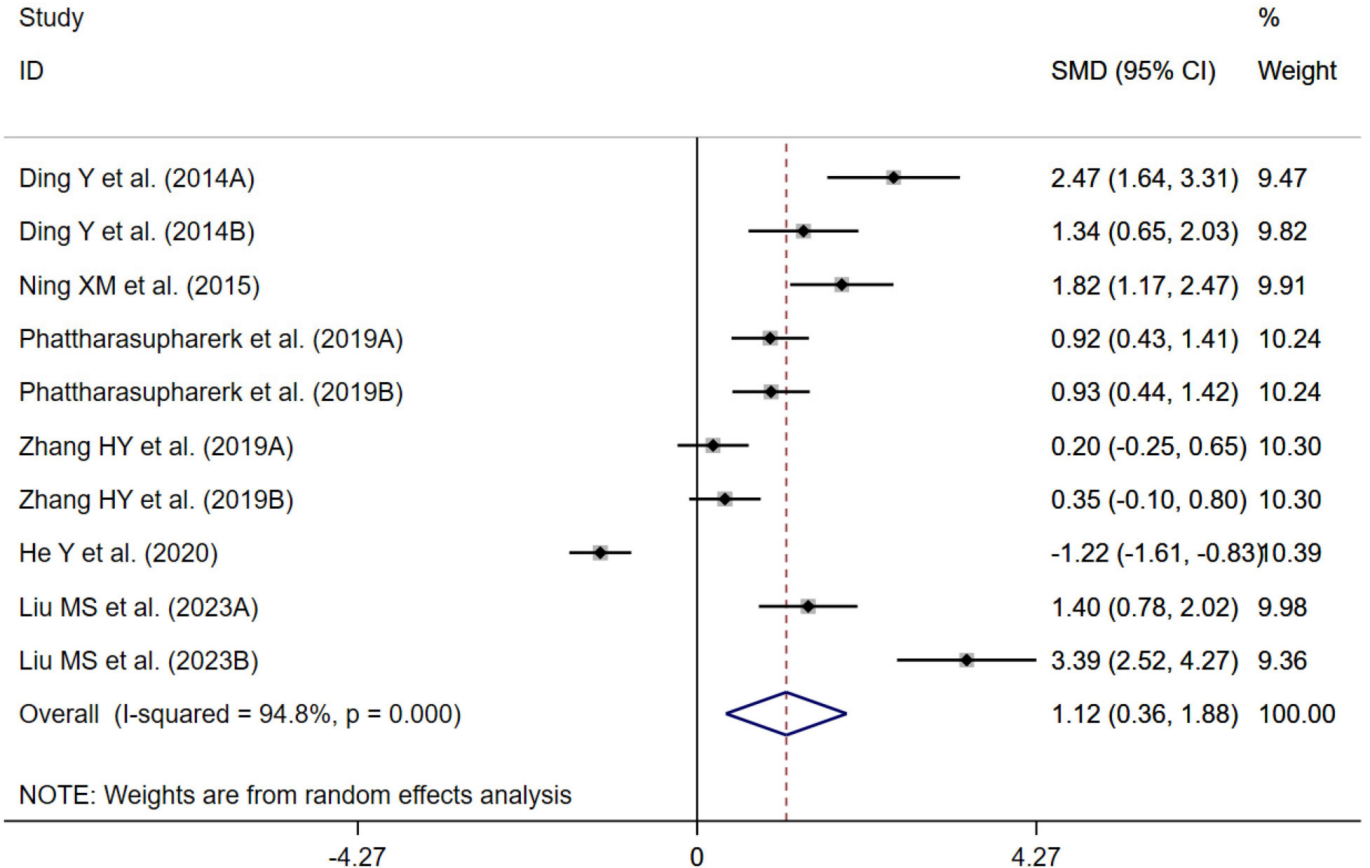
Forest plot of the spine range of motion. In Ding and Wang (23), two assessment methods were adopted to evaluate proprioception. In Phattharasupharerk et al. (35), two assessment methods were adopted to evaluate proprioception. In Zhang (38), two assessment methods were adopted to evaluate proprioception. In Liu et al. (52), two assessment methods were adopted to evaluate proprioception.

6 randomized controlled trials (23, 28, 35, 38, 39, 52) were evaluated to assess the impact of traditional Chinese exercises on spine range of motion associated with nonspecific low back pain. A random-effects model meta-analysis revealed that traditional Chinese exercises improved spine range of motion compared to the control group [SMD = 1.12; 95% CI (0.36, 1.88); *P* = 0.004; *I*^2^ = 94.8%, *P* < 0.001]. The difference was not statistically significant, but it suggests a potential clinical benefit worthy of further investigation (Figure 9).

#### 3.4.7 Subgroup analysis

Subgroup analyses were conducted based on disease duration, age, specific types of traditional Chinese exercise, sex, and treatment duration to explore potential sources of heterogeneity in pain, ODI, and effective rate. The results are presented in Supplementary Material S3.

#### 3.4.8 Pain intensity

Subgroup analyses based on pain duration, age, type of traditional Chinese exercise, sex, and treatment duration were performed to evaluate pain relief as measured by the Visual Analogue Scale (VAS). No significant heterogeneity was detected across subgroups. Nonetheless, traditional Chinese exercises were consistently associated with statistically significant reductions in VAS pain scores in all subgroup analyses.

#### 3.4.9 ODI

Subgroup analyses based on pain duration, age, type of traditional Chinese exercise, sex, and treatment duration were performed for the Oswestry Disability Index (ODI), and no sources of heterogeneity were identified. Traditional Chinese exercises were found to significantly reduce the risk of functional disability across all subgroups examined, including pain duration (<3 years,≥3 years, other categories), age (<45 years,≥45 years), sex (>50% female, >50% male, other categories), treatment duration (0–1 month, 1–3 months, 3–9 months), and different types of traditional Chinese exercise.

#### 3.4.10 Treatment effective rate

Subgroup analyses based on pain duration, age, type of traditional Chinese exercise, sex, and treatment duration were performed for the response rate, and no sources of heterogeneity were identified. Traditional Chinese exercises were found to increase the treatment effective rate across all subgroups examined, including pain duration (<3 years, ≥3 years), age (<45 years, ≥45 years), different types of traditional Chinese exercise, and sex (>50% female, >50% male, other categories).

#### 3.4.11 Sensitivity analysis

Sensitivity analyses were performed for all outcome indicators using a leave-one-out method to assess the influence of individual studies on the pooled results. The findings indicated that none of the included studies significantly affected the overall combined effect sizes, suggesting that the results of this meta-analysis are generally robust and reliable. The results of the sensitivity analyses are presented in Supplementary Material S4.

#### 3.4.12 Publication bias

To ensure the validity of the meta-analysis results, funnel plots, Egger's test, and Begg's test were employed to detect publication bias for the primary outcome indicators. The results indicated no significant publication bias for the ODI (*P* = 0.819) or the treatment response rate (*P* = 0.327). In contrast, significant publication bias was detected for Pain Intensity (*P* < 0.01). After applying the trim-and-fill method, which imputed 9 additional studies, the conclusions remained unchanged, further confirming the robustness of the findings.

## 4 Discussion

### 4.1 Research results

Our study demonstrates that traditional Chinese exercise yields significant multifaceted improvements in patients with nonspecific low back pain. In terms of pain intensity, traditional Chinese exercise significantly reduced pain. Li et al. reported that Baduanjin had a positive effect on reducing pain in patients with discogenic low back pain compared with conventional exercise (12); Zhang et al. also reported that traditional exercises such as Tai Chi and Qigong significantly improved pain intensity in patients with low back pain (13). Despite the statistically significant improvement shown in the pooled analysis, the presence of heterogeneity across studies calls for cautious interpretation of this result. For example, in previous observational studies, the intervention frequency of Baduanjin varied, as did the intervention duration. This protocol-level diversity may render the pooled effect size difficult to translate directly into unified clinical recommendations. Subgroup analyses suggested that there were numerical differences in pain improvement across subgroups stratified by disease duration, age, type of exercise, sex, and treatment duration. No particular subgroup was identified to have significantly superior pain relief. This may be attributed to the fact that patients generally possess relatively sufficient vital energy (referred to as “zhengqi” in TCM), which contributes to the analgesic effect of traditional Chinese exercises. However, it should be noted that this inference is primarily derived from theoretical interpretations of TCM and remains to be substantiated by direct biological evidence. The causal relationship between disease duration and treatment efficacy still requires further validation through additional high-quality studies. Among the various types of exercise, Liuzijue demonstrated the most significant effect in alleviating pain. Liu Zi Jue, a traditional breathing exercise, emphasizes coordinated breath regulation with slow, gentle movements, which may enhance parasympathetic tone, reduce stress-induced muscle tension, and improve diaphragmatic function, thereby alleviating low back pain (59). Regarding the Oswestry Disability Index (ODI), TCE can effectively reduce the degree of patient dysfunction. Zhang et al. indicated that similar exercise interventions have a positive effect on improving lumbar function (60). Among different exercise types, the Liuzijue group demonstrated the most substantial improvement in reducing ODI; in terms of treatment duration, short-term interventions produced more pronounced effects in lowering ODI. Regarding the overall treatment effective rate, traditional Chinese exercise was found to improve the effective rate. Subgroup analysis revealed that disease duration, age, sex, and treatment duration had varying effects on the effective rate. Patients with a disease duration of less than 3 years showed a more pronounced improvement in response rate. Both patients under 45 years of age and those aged 45 or older exhibited improved response rates. When the treatment duration was ≤1 month, a marked increase in response rate was noted with the outcomes consistent with previous findings. However, the reliability of the “effective rate” outcome itself remains contentious. Among the trials included in this study, the criteria for determining therapeutic efficacy were not standardized: some employed reductions in pain scores as the benchmark, while others relied on patients' self-reported “marked improvement.” This inconsistency in outcome definitions may have inflated the credibility of the pooled effect size. Sensitivity analysis revealed that after excluding the study by Xu et al., heterogeneity shifted from significant to non-significant, indicating that a single study exerted considerable influence on the overall conclusion—a finding that further underscores the fragility of the current evidence base.

### 4.2 Analysis of results

TCE can improve spinal stability and core muscle strength. One of the core issues of NLBP is the decline in spinal stability, which is closely related to the weakened strength of the core muscle group (61). Recent studies have revealed that traditional Chinese sports, through specific movement training, can enhance the strength of the core muscle groups in the waist and abdomen, improve the stability of the spine, and reduce pain caused by spinal instability (62). For instance, Tai Chi can enhance the muscle strength and bone density of practitioners, improve their motor and balance functions, and alleviate clinical symptoms (63). Some specific movements in Baduanjin can also improve the contraction and relaxation ability of the erector spinae muscles and enhance the stability of the lumbar vertebrae through the forward and backward flexion of the waist, thereby alleviating chronic low back pain (42). A RCT conducted by Liu et al. indicated that progressive postural control exercises and core stabilization exercises markedly improved pain and muscle function in young adults with chronic nonspecific low back pain. The underlying mechanisms could be associated with neuromuscular plasticity and adaptive adjustments (64). It should be specifically emphasized that the discussion regarding mechanisms of action is primarily based on the synthesis and analysis of existing literature rather than data directly generated by this study. Although we attempted to elucidate potential pathways through which traditional mind-body exercises alleviate nonspecific low back pain from multiple dimensions—including biomechanical, neurophysiological, and psychosocial perspectives—these mechanistic explanations remain exploratory hypotheses involving a certain degree of inferential extrapolation. For instance, while multiple studies support the association between Tai Chi and improved core muscle strength relevant to spinal stability (65), the causal relationship between pain relief and enhanced muscular function has yet to be confirmed through rigorously designed mechanistic validation trials. Similarly, discussions regarding mechanisms such as inflammatory marker modulation and neuroplasticity changes are largely derived from studies conducted in healthy populations or other disease groups, and their reproducibility in patients with nonspecific low back pain remains to be validated.

In fact, TCE exert positive effects on both blood circulation and neural regulation. Wang et al. conducted a case-control study to investigate the impact of long-term TCC practice on cutaneous microcirculatory function and nitric oxide metabolism in healthy elderly males. The results demonstrated that prolonged TCC practice can enhance nitric oxide-mediated endothelium-dependent vasodilation, thereby improving cutaneous microcirculatory perfusion and thermal regulatory capacity, all of which were associated with phenotypes such as the upregulation of endothelial nitric oxide synthase expression mediated by blood flow and enhanced vascular reactivity (66). Recent advances in understanding intervertebral disc degeneration have highlighted the critical role of cartilage endplate cells (CEPCs) in maintaining disc health and the pathological significance of inflammatory degeneration of CEPCs. Zhan et al. demonstrated that targeted delivery of engineered exosomes loaded with salvianolic acid A to CEPCs effectively reduced intracellular iron ion influx and reactive oxygen species, inhibited the mtDNA-cGAS-STING pathway, and alleviated CEPC inflammation (67). Furthermore, their composite hydrogel system neutralized excess hydrogen ions in the acidic microenvironment, thereby directly mitigating nucleus pulposus cell inflammation and senescence. These findings provide mechanistic insights at the cellular and molecular levels that may help explain how traditional Chinese exercises exert therapeutic effects through modulating inflammatory cascades and improving the intervertebral disc microenvironment. Similar report also claimed that long-term practice of Tai Chi could reduce inflammatory factors such as TNF-α and IL-6, down-regulate inflammatory risk markers, and alleviate the risk level of inflammation-related comorbidities. These changes are significantly associated with pain relief (68). Furthermore, recent evidence suggests that obesity-related anthropometric indicators, particularly the weight-adjusted waist index (WWI), are significantly associated with chronic low back pain risk, with WWI demonstrating stronger predictive value than traditional BMI (15). This finding underscores the potential importance of body composition—especially the balance between fat and muscle mass—in the pathogenesis of CLBP, and may provide a complementary perspective for understanding how TCE exert therapeutic effects through improving body composition and metabolic profiles. However, it is worth noting that the aforementioned studies were predominantly conducted in healthy populations, and their reproducibility in patients with nonspecific low back pain remains to be validated.

Furthermore, TCE have been widely demonstrated to be beneficial to psychological and emotional well-being. Patients with nonspecific low back pain often experience anxiety and depression. Jiao et al. found that patients subjected to Baduanjin therapy showed significant improvement in depressive symptoms. The data indicated that Baduanjin enhances emotion regulation through mind–body integration training, potentially by modulating neuroendocrine responses and improving pain self-efficacy (69). Song et al. also reported in clinical practice the positive impact of modified Tai Chi on psychological states. Their research found that Tai Chi, as a low-intensity physical and mental exercise, can bring similar emotional regulation benefits to patients with chronic low back pain by enhancing body perception, promoting the release of endogenous endorphins, and improving neural plasticity (70). Ungkarat et al. confirmed that Tai Chi can improve cognitive function by upregulating BDNF levels. Specifically, traditional mind-body exercises such as Tai Chi may relief low back pain and related psychological issues through mechanisms like enhancing the regulatory capacity of the central nervous system and promoting emotional regulation pathways. This “mind-body” synergy model may more effectively induce the expression of neurotrophic factors, providing a potential non-pharmaceutical intervention approach for the midweek nerve regulation of patients with chronic low back pain (71).

### 4.3 Limitations and prospects

This study has several limitations. First, the majority of the included studies were conducted in China, which limits the generalizability of the findings to other populations. Chinese patients may show greater responsiveness to traditional mind-body exercises due to cultural familiarity with practices like Tai Chi and Baduanjin, potentially producing effect sizes that exceed what might be observed in populations without such embedded traditions (72). Second, as the intervention group received exercise-based treatments while the control group mostly received usual care or conventional treatment, blinding of participants and personnel posed challenges. Third, variations existed across the included studies in terms of the specific interventions administered, which encompassed multiple types of TCE, as well as differences in exercise intensity, frequency, and duration. Additionally, heterogeneity was introduced due to differences in population characteristics—such as age, sex, and disease duration—as well as inconsistencies in measurement tools and evaluation criteria across studies. Although subgroup analyses were conducted to explore potential sources of heterogeneity, they could not fully account for the observed variations. Fourth, discrepancies in the assessment methods and outcome measures used across studies may have affected the consistency and reliability of the results. Fifth, none of the included studies classified patients according to TCM syndrome patterns (e.g., cold-dampness syndrome, kidney deficiency syndrome). Therefore, it was not possible to evaluate the differential effects of the interventions based on TCM syndrome differentiation. However, although this study confirmed the overall therapeutic efficacy, significant heterogeneity and methodological limitations remain. Future research should focus on conducting large-sample RCTs with rigorous methodology, employing standardized exercise intervention protocols—such as frequency, intensity, and duration. Furthermore, in-depth exploration of the underlying mechanisms is warranted. This should incorporate multidisciplinary technologies such as modern imaging (e.g., fMRI), biomechanical analysis, and serum inflammatory factor detection to elucidate the therapeutic basis from multidimensional perspectives encompassing neural, muscular, psychological, and immune dimensions. Additionally, personalized exercise prescriptions based on TCM syndrome differentiation or modern medical classifications should be explored to achieve more precise rehabilitation therapy.

Despite confirming the overall benefits of traditional Chinese exercise, this review has limitations that warrant careful consideration. The generalizability of our findings is constrained by the geographical concentration of the included trials, most of which were conducted within China. Methodologically, the inherent nature of exercise interventions presented challenges in blinding participants and personnel, potentially introducing performance bias. A more substantial source of heterogeneity stems from the considerable variations across studies, not only in the specific exercise modalities employed but also in their intensity, frequency, and duration, compounded by differences in participant demographics and the tools used to assess outcomes. Although subgroup analyses were undertaken, they could not fully resolve this heterogeneity. Furthermore, the inability to incorporate the principle of TCM syndrome differentiation is a crucial point that needs to be improved, as none of the primary studies classified patients according to TCM patterns (e.g., cold-dampness, kidney deficiency), precluding an analysis of personalized therapeutic effects. To advance the field, future research could prioritize large-scale, rigorously designed randomized controlled trials that implement standardized intervention protocols. A deeper investigation into the underlying mechanisms is also significant, leveraging multidisciplinary approaches such as functional magnetic resonance imaging (fMRI), biomechanical analysis, and biomarker assays to elucidate effects across neural, muscular, immune, and psychological domains. Ultimately, developing personalized exercise prescriptions grounded in TCM syndrome differentiation or modern medical classifications holds promise for achieving more precise and effective rehabilitation.

## 5 Conclusion

This meta-analysis, synthesizing evidence from 38 randomized controlled trials, demonstrates that traditional Chinese exercises are an effective, safe, and economical non-pharmacological therapy for the management of nonspecific low back pain. Specifically, these traditional exercise therapies demonstrated significant efficacy in the short term: just one month of regular practice significantly reduced pain intensity [RR = −0.90; 95% CI (−1.23, −0.57); *P* < 0.001] and improved lumbar dysfunction [WMD = −5.96; 95% CI (−8.51, −3.42); *P* < 0.001]. It not only significantly alleviates pain, improves functional disability and quality of life, but also increases the overall treatment response rate. This study provides key evidence to support the stepped-care approach for NLBP management. Traditional Chinese exercise is characterized by rapid onset of benefits, high safety, and good patient acceptance, making it particularly suitable for elderly patients, those sensitive to adverse drug reactions, or individuals inclined toward holistic health management. Our study aims to promote the integration of TCE as an important supplement or suitable alternative into conventional treatments for NLBP, such as physical therapy and health education, thereby providing evidence-based medical support. By promoting these exercise therapies deeply rooted in Chinese culture, not only can the disease burden on patients be effectively alleviated, but it also holds profound significance for reducing healthcare economic costs and advancing the modernization and internationalization of TCM.

## Data Availability

The datasets presented in this study can be found in online repositories. The names of the repository/repositories and accession number(s) can be found in the article/Supplementary Material.
