# Supplementary material for: Moving for relief: a meta-analysis on traditional Chinese exercise and nonspecific low back pain
Source: Front Pain Res (Lausanne). 2026 Apr 20;7:1729225. doi: 10.3389/fpain.2026.1729225 (PMC13136288; doi:10.3389/fpain.2026.1729225)
Supplement: Supplementary file 1 [file Datasheet1.zip › Supplementary Material/Supplementary Material 3 Subgroup Analysis Chart.docx]

**亚组分析图表**

| Outcomes | Subgroups | Number of studies | ES(95%CI) | Heterogeneity | | Regression model |
| --- | --- | --- | --- | --- | --- | --- |
|  |  |  |  | I^2^ | P | P |
| Pain rating | Disease duration | | | | | |
|  | ＜3 years | 13 | -1.42  （-1.79，-1.05） | 84.0% | 0.000 | 0.386 |
|  | ≥3 years | 8 | -0.96  （-1.25，-0.67） | 66.4% | 0.004 |  |
|  | Age | | | | | |
|  | ＜60 years old | 25 | -1.52  （-1.84，-1.20） | 89.6% | 0.000 | 0.679 |
|  | ≥60 years old | 6 | -0.88  （-1.25，-0.67） | 70.7% | 0.004 |  |
|  | Traditional sports types | | | | | |
|  | Tai Chi | 11 | -1.96  （-2.53，-1.38） | 91.7% | 0.000 | 0.148 |
|  | Baduanjin | 12 | -1.24  （-1.60，-0.89） | 82.6% | 0.000 |  |
|  | Wu Qin Xi | 5 | -1.03  （-1.73，-0.33） | 90.0% | 0.000 |  |
|  | Liuzi jue | 3 | -1.56  （-2.19，-0.93） | 65.4% | 0.055 |  |
|  | Yijingjin | 1 | -1.74  （-2.40，-1.09） | - | - |  |
|  | Qigong | 3 | -0.89  （-1.87，-0.09） | 90.1% | 0.000 |  |
|  | Sex | | | | | |
|  | Female＞50% | 15 | -1.57  （-2.06，-1.09） | 92.5% | 0.000 | 0.550 |
|  | Male＞50% | 13 | -1.32  （-1.73，-0.92） | 87.5% | 0.000 |  |
|  | Treatment Duration | | | | | |
|  | 0-1 months | 9 | -1.13  （-1.45，-0.81） | 68.7% | 0.001 | 0.806 |
|  | 1-3 months | 22 | -1.64  （-2.03，-1.25） | 91.4% | 0.000 |  |
|  | 3-9 months | 4 | -1.02  （-1.48，-0.56） | 73.7% | 0.000 |  |
| ODI | Disease duration | | | | | |
|  | ＜3 years | 10 | -9.11  （-12.47，-5.74） | 94.8% | 0.000 | 0.676 |
|  | ≥3 years | 5 | -3.81  （-5.25，-2.37） | 62.5% | 0.030 |  |
|  | Age | | | | | |
|  | ＜50 years old | 13 | -9.61  （-12.26，-6.95） | 92.2% | 0.000 | 0.012 |
|  | ≥50 years old | 6 | -3.81  （-5.25，-2.37） | 55.4% | 0.047 |  |
|  | Traditional sports types | | | | | |
|  | Baduanjin | 10 | -5.15  （-6.79，-3.51） | 71.3% | 0.000 | 0.646 |
|  | Wu Qin Xi | 2 | -5.30  （-6.40，-4.21） | 0.0% | 0.577 |  |
|  | Liuzi jue | 3 | -12.34  （-13.71，-10.97） | 0.0% | 0.392 |  |
|  | Tai Chi | 2 | -13.51  （-26.05，-0.98） | 98.1% | 0.000 |  |
|  | Yijingjin | 2 | -6.69  （-9.74，-3.65） | 0.0% | 0.571 |  |
|  | Sex | | | | | |
|  | Female＞50% | 6 | -5.04  （-7.69，-2.39） | 76.4% | 0.001 | 0.143 |
|  | Male＞50% | 8 | -8.24  （-11.69，-4.79） | 95.8% | 0.000 |  |
|  | Treatment Duration | | | | | |
|  | ＜1 month | 10 | -10.55  （-13.94，-7.16） | 93.1% | 0.000 | 0.098 |
|  | 1-3 months | 9 | -4.75  （-6.13，-3.37） | 67.4% | 0.002 |  |
| Effective rate | Disease duration | | | | | |
|  | ＜3 years | 4 | 1.42  （1.21,1.66） | 43.9% | 0.148 | 0.456 |
|  | ≥3 years | 2 | 1.21  （1.08，1.36） | 0.0% | 0.632 |  |
|  | Age | | | | | |
|  | ＜35 years old | 3 | 1.44  （1.20，1.72） | 17.0% | 0.300 | 0.258 |
|  | ≥35 years old | 5 | 1.25  （1.13，1.37） | 0.0% | 0.652 |  |
|  | Traditional sports types | | | | | |
|  | Baduanjin | 3 | 1.44  （1.20，1.72） | 17.0% | 0.300 | 0.420 |
|  | Liuzi jue | 2 | 1.21  （1.02，1.42） | 0.0% | 0.634 |  |
|  | Sex | | | | | |
|  | Female＞50% | 2 | 1.31  （1.09，1.57） | 0.0% | 0.750 | 0.594 |
|  | Male＞50% | 3 | 1.22  （1.10，1.36） | 0.0% | 0.849 |  |
|  | Treatment Duration | | | | | |
|  | ≤3 weeks | 2 | 1.42  （1.21，1.66） | 0.0% | 0.585 | 0.023 |
|  | ＞3 weeks | 6 | 1.21  （1.08，1.36） | 0.0% | 0.984 |  |
