# Supplementary material for: Moving for relief: a meta-analysis on traditional Chinese exercise and nonspecific low back pain
Source: Front Pain Res (Lausanne). 2026 Apr 20;7:1729225. doi: 10.3389/fpain.2026.1729225 (PMC13136288; doi:10.3389/fpain.2026.1729225)
Supplement: Supplementary file 1 [file Datasheet1.zip › Supplementary Material/Supplementary Material 1 Literature search strategy.docx]

# Supplementary Text 1 Literature search strategy

**1.Pubmed**

| Search number | Query |
| --- | --- |
| #1 | "Low Back Pain"[Mesh] |
| #2 | ((((((((((((((Low Back Pain*[Title/Abstract]) OR (Low Back Ache*[Title/Abstract])) OR (Low Back*[Title/Abstract])) OR (lowback pain[Title/Abstract])) OR (Lower Back Pain*[Title/Abstract])) OR (Lumbago[Title/Abstract])) OR (lumba*[Title/Abstract])) OR (chronic back pain[Title/Abstract])) OR (backache[Title/Abstract])) OR (dorsalgia[Title/Abstract])) OR (LBP[Title/Abstract])) OR (loin pain[Title/Abstract])) OR (low backpain[Title/Abstract])) OR (lumbodynia[Title/Abstract])) OR (lumbosacr*[Title/Abstract]) |
| #3 | ("Low Back Pain"[Mesh]) OR (((((((((((((((Low Back Pain*[Title/Abstract]) OR (Low Back Ache*[Title/Abstract])) OR (Low Back*[Title/Abstract])) OR (lowback pain[Title/Abstract])) OR (Lower Back Pain*[Title/Abstract])) OR (Lumbago[Title/Abstract])) OR (lumba*[Title/Abstract])) OR (chronic back pain[Title/Abstract])) OR (backache[Title/Abstract])) OR (dorsalgia[Title/Abstract])) OR (LBP[Title/Abstract])) OR (loin pain[Title/Abstract])) OR (low backpain[Title/Abstract])) OR (lumbodynia[Title/Abstract])) OR (lumbosacr*[Title/Abstract])) |
| #4 | "Tai Ji"[Mesh] |
| #5 | ((((((((((((((Tai Chi Chinese exercise*[Title/Abstract]) OR (wushu[Title/Abstract])) OR (martial art[Title/Abstract])) OR (Taijiquan[Title/Abstract])) OR (Tai Chi[Title/Abstract])) OR (Tai Ji[Title/Abstract])) OR (Baduanjin[Title/Abstract])) OR (Eight-Section Brocade[Title/Abstract])) OR (Chi Kung[Title/Abstract])) OR (Qigong[Title/Abstract])) OR (qi gong[Title/Abstract])) OR (Yi jing jin[Title/Abstract])) OR (Wu Qin Xi[Title/Abstract])) OR (Five Animal Frolics[Title/Abstract])) OR (Liuzijue[Title/Abstract]) |
| #6 | ("Tai Ji"[Mesh]) OR (((((((((((((((Tai Chi Chinese exercise*[Title/Abstract]) OR (wushu[Title/Abstract])) OR (martial art[Title/Abstract])) OR (Taijiquan[Title/Abstract])) OR (Tai Chi[Title/Abstract])) OR (Tai Ji[Title/Abstract])) OR (Baduanjin[Title/Abstract])) OR (Eight-Section Brocade[Title/Abstract])) OR (Chi Kung[Title/Abstract])) OR (Qigong[Title/Abstract])) OR (qi gong[Title/Abstract])) OR (Yi jing jin[Title/Abstract])) OR (Wu Qin Xi[Title/Abstract])) OR (Five Animal Frolics[Title/Abstract])) OR (Liuzijue[Title/Abstract])) |
|  |  |

**2.Cochrane**

| Search number | Query |
| --- | --- |
| #1 | MeSH descriptor: [Low Back Pain] explode all trees |
| #2 | (Low Back Pain*):ti,ab,kw OR (Low Back Ache*):ti,ab,kw OR (Low Back*):ti,ab,kw OR (lowback pain):ti,ab,kw OR (Lower Back Pain*):ti,ab,kw |
| #3 | (Lumbago):ti,ab,kw OR (lumba*):ti,ab,kw OR (chronic back pain):ti,ab,kw OR (backache):ti,ab,kw OR (dorsalgia):ti,ab,kw |
| #4 | (LBP):ti,ab,kw OR (loin pain):ti,ab,kw OR (low backpain):ti,ab,kw OR (lumbodynia):ti,ab,kw OR (lumbosacr*):ti,ab,kw |
| #5 | #1 OR #2 OR #3 OR #4 |
| #6 | MeSH descriptor: [Tai Ji] explode all trees |
| #7 | (Tai Chi Chinese exercise*):ti,ab,kw OR (wushu):ti,ab,kw OR (martial art):ti,ab,kw OR (Taijiquan):ti,ab,kw OR (Tai Chi):ti,ab,kw |
| #8 | (Tai Ji):ti,ab,kw OR (Baduanjin):ti,ab,kw OR (Eight-Section Brocade):ti,ab,kw OR (Chi Kung):ti,ab,kw OR (Qigong):ti,ab,kw |
| #9 | (qi gong):ti,ab,kw OR (Yi jing jin):ti,ab,kw OR (Wu Qin Xi):ti,ab,kw OR (Five Animal Frolics):ti,ab,kw OR (Liuzijue):ti,ab,kw |
| #10 | #6 OR #7 OR #8 OR #9 |
| #11 | #5 AND #10 |

**3.Embase**

| Search number | Query |
| --- | --- |
| #1 | 'tai chi'/exp |
| #2 | 'tai chi chinese exercise*':ab,ti OR wushu:ab,ti OR 'martial art':ab,ti OR taijiquan:ab,ti OR 'tai chi':ab,ti OR 'tai ji':ab,ti OR baduanjin:ab,ti OR 'eight-section brocade':ab,ti OR 'chi kung':ab,ti OR qigong:ab,ti OR 'qi gong':ab,ti OR 'yi jing jin':ab,ti OR 'wu qin xi':ab,ti OR 'five animal frolics':ab,ti OR liuzijue:ab,ti |
| #3 | #1 OR #2 |
| #4 | 'low back pain'/exp |
| #5 | 'low back pain*':ab,ti OR 'low back ache*':ab,ti OR 'low back*':ab,ti OR 'lowback pain':ab,ti OR 'lower back pain*':ab,ti OR lumbago:ab,ti OR lumba*:ab,ti OR 'chronic back pain':ab,ti OR backache:ab,ti OR dorsalgia:ab,ti OR lbp:ab,ti OR 'loin pain':ab,ti OR 'low backpain':ab,ti OR lumbodynia:ab,ti OR lumbosacr*:ab,ti |
| #6 | #4 OR #5 |
| #7 | #3 AND #6 |

**4.Web of science**

| Search number | Query |
| --- | --- |
| #1 | Low Back Pain* (Topic) OR Low Back Ache* (Topic) OR Low Back* (Topic) OR lowback pain (Topic) OR Lower Back Pain* (Topic) OR Lumbago (Topic) OR lumba* (Topic) OR chronic back pain (Topic) OR backache (Topic) OR dorsalgia (Topic) OR LBP (Topic) OR loin pain (Topic) OR low backpain (Topic) OR lumbodynia (Topic) OR lumbosacr* (Topic) |
| #2 | Tai Chi Chinese exercise* (Topic) OR wushu (Topic) OR martial art (Topic) OR Taijiquan (Topic) OR Tai Chi (Topic) OR Tai Ji (Topic) OR Tai Ji (Topic) OR Eight-Section Brocade (Topic) OR Chi Kung (Topic) OR Qigong (Topic) OR qi gong (Topic) OR Yi jing jin (Topic) OR Wu Qin Xi (Topic) OR Five Animal Frolics (Topic) OR Liuzijue (Topic) |
| #3 | #1 AND #2 |

**5. The following table shows an example of a search strategy for Chinese databases (Sinomed).**

| Search number | Query |
| --- | --- |
| #1 | "Low back pain"[ Unweighted: extended] |
| #2 | "Tai Ji"[ Unweighted: extended] OR "Tai Chi"[ Unweighted: extended] |
| #3 | ( "Traditional sports"[Common field: Intelligence] OR "Chinese sports"[Common field: Intelligence] OR "Wushu"[Common field: Intelligence] OR "Sanda"[Common field: Intelligence] OR "Qigong"[Common field: Intelligence] OR "Five-animal exercises"[Common field: Intelligence] OR "Tai Ji"[Common field: Intelligence] OR "Baduanjin"[Common field: Intelligence] OR "Yijingjin"[Common field: Intelligence] OR "Liuzi jue"[Common field: Intelligence]) |
| #4 | ( "Non-specific low back pain"[Common field: Intelligence] OR " Low back pain "[Common field: Intelligence] OR "Lower back pain"[Common field: Intelligence] OR "Lumbago and backache"[Common field: Intelligence] OR " low discomfort "[Common field: Intelligence]) |
| #5 | (( "Non-specific low back pain"[Common field: Intelligence] OR " Low back pain "[Common field: Intelligence] OR "Lower back pain"[Common field: Intelligence] OR "Lumbago and backache"[Common field: Intelligence] OR " low discomfort "[Common field: Intelligence])) OR ("Low back pain "[ Unweighted: extended]) |
| #6 | (( "Traditional sports"[Common field: Intelligence] OR "Chinese sports"[Common field: Intelligence] OR "Wushu"[Common field: Intelligence] OR "Sanda"[Common field: Intelligence] OR "Qigong"[Common field: Intelligence] OR "Five-animal exercises"[Common field: Intelligence] OR "Tai Ji"[Common field: Intelligence] OR "Baduanjin"[Common field: Intelligence] OR "Yijingjin"[Common field: Intelligence] OR "Liuzi jue"[Common field: Intelligence])) OR ("Tai Ji"[ Unweighted: extended] OR "Tai Chi"[ Unweighted: extended]) |
| #7 | ((( "Traditional sports"[Common field: Intelligence] OR "Chinese sports"[Common field: Intelligence] OR "Wushu"[Common field: Intelligence] OR "Sanda"[Common field: Intelligence] OR "Qigong"[Common field: Intelligence] OR "Five-animal exercises"[Common field: Intelligence] OR "Tai Ji"[Common field: Intelligence] OR "Baduanjin"[Common field: Intelligence] OR "Yijingjin"[Common field: Intelligence] OR "Liuzi jue"[Common field: Intelligence])) OR ("Tai Ji"[ Unweighted: extended] OR "Tai Chi"[ Unweighted: extended])) AND ((( "Non-specific low back pain"[Common field: Intelligence] OR " Low back pain "[Common field: Intelligence] OR "Lower back pain"[Common field: Intelligence] OR "Lumbago and backache"[Common field: Intelligence] OR " low discomfort "[Common field: Intelligence])) OR ("Low back pain "[ Unweighted: extended])) |
